# Antimicrobial Sensitivity Patterns of Bacteria Causing Urinary Tract Infections: A Retrospective Study of Elderly Patients Admitted to a Tertiary Care Hospital in Bhubaneswar, India

**DOI:** 10.7759/cureus.77399

**Published:** 2025-01-13

**Authors:** Basanti Kumari Pathi, Smrutisree Mohapatra, Vibha Sharma, Ipsa Mohapatra, Kumudini Panigrahi, Shubhransu Patro

**Affiliations:** 1 Department of Microbiology, Kalinga Institute of Medical Sciences, Bhubaneswar, IND; 2 Department of General Medicine, Kalinga Institute of Medical Sciences, Bhubaneswar, IND; 3 Department of Community Medicine, Kalinga Institute of Medical Sciences, Bhubaneswar, IND

**Keywords:** antibiotic sensitivity, elderly patients, escherichia coli, ipd, opd, uti

## Abstract

Background: The aging population faces various health challenges, which has increased the burden on healthcare systems. Elderly individuals are more susceptible to urinary tract infections (UTIs), and rising bacterial resistance to antibiotics is a significant global issue.

Objectives: This study compared the bacterial etiology and antibiotic sensitivity testing (AST) patterns of bacteria causing UTIs in elderly patients attending a tertiary care hospital across the intensive care unit (ICU), inpatient department (IPD), and outpatient department (OPD) and analyzed the association of the UTI with the presence of comorbidities and outcomes in the study population.

Methodology: This retrospective study was conducted at the Department of Microbiology at a tertiary care hospital in Bhubaneswar. The demographic, clinical, and microbiological data were collected using a data abstraction form from the electronic medical record from January to December 2022. The study relied entirely on previously documented results. The urine samples received in the laboratory were processed following standard protocol. Identification and AST were done in the Vitek-2 (bioMérieux, Marcy-l'Étoile, France) automated system. AST was interpreted following the Clinical and Laboratory Standards Institute 2021. The Epi Info statistical software, version 7.2.3.1 (Centers for Disease Control and Prevention, Atlanta, GA), was used to analyze the data after loading it into a Microsoft Excel spreadsheet (Microsoft Corporation, Redmond, WA). The results were displayed as mean and standard deviation, frequencies, and percentages. A p value of less than 0.05 is considered statistically significant. Analysis of variance, chi-square test, and Fisher's exact test were employed as association tests.

Results: A total of 4,030 urine samples were processed for culture and sensitivity testing from the study population from January to December 2022. The data were retrospectively compiled and analyzed. The majority of samples (1,600; 39.70%) were received from the ICU, followed by IPD (1,255; 31.14%), and OPD (1,175; 29.15%). Culture positivity was 1,560 (38.70%). The mean age of patients was 69.74 ± 7.52 years, ranging from 60 to 97 years. Males, 2,559 (63.50%), reported more UTIs than females. Chronic kidney disease (CKD, p = 0.0269) and hypertension (HTN, p = 0.0058), associated with UTI, were statistically significant. The most common bacterial isolate was *Escherichia coli at* 39.67% (619/1,560), followed by *Klebsiella* spp., 323 (20.70%). *E. coli* was most sensitive to amikacin (269/567 in OPD, 165/567 in IPD, and 133/567 in ICU). *Staphylococcus aureus* isolates from OPD patients were more sensitive to antibiotics than those from ICU and IPD patients. *Klebsiella* spp. was mainly sensitive to ertapenem, tigecycline, and gentamicin. *Enterococcus faecalis* isolates were mainly sensitive to tigecycline, linezolid, teicoplanin, and vancomycin.

Conclusion: The study showed that UTI was more prevalent in 60-69-year-old people and men. *E. coli* was the most common among OPD, IPD, and ICU elderly patients. Bacterial isolates from OPD patients were more sensitive to antibiotics than IPD and ICU patients. Patients mostly suffering from different comorbidities like HTN and CKD comprised a higher proportion of the ICU patients. All mortality of patients reported was from ICU.

## Introduction

With an increase in life expectancy and accessibility to better healthcare services, there has been a rise in the aging population worldwide; their varied group of health problems pose a significant healthcare burden. The elderly are at higher risk of urinary tract infections (UTIs), accounting for one of the most common causes of hospitalization due to bacterial infections [1]. In contrast to younger adults, older persons have difficulty differentiating between asymptomatic bacteriuria (ASB) and symptomatic UTI. An infection of the urinary system is commonly referred to as a UTI, and it can affect either the upper or lower urinary tract, depending on the anatomical structure [2]. The presence of symptoms unique to the urinary tract combined with a quantitative count of 10,000,00 colony-forming units (CFUs) of bacteria per milliliter in a single urine specimen is typically required to qualify as a symptomatic UTI [3,4]. Uncomplicated and complicated UTIs are the two clinical subtypes. Healthy people without neurological or structural urinary tract problems, such as cystitis or pyelonephritis, are primarily affected by uncomplicated UTIs. Urinary obstruction, neurological disorders that cause retention of urine, renal failure, renal transplantation, pregnancy, and the presence of foreign bodies like calculi, indwelling catheters, or other drainage devices are among the factors that compromise the urinary tract and cause complicated UTIs [5].

UTIs cause around 25% of all infections in older adults each year, accounting for over seven million hospital visits, one million ER visits, and 100,000 hospitalizations without any structural or neurological causes [6]. The overall incidence of UTI among older men and women ranges from one infection per 14-20 persons-years. The incidence of UTI was 0.07 per person-year in a large prospective cohort study of postmenopausal women living in the community and 0.12 per person-year in older women with diabetes [7]. For men aged 65-74, the incidence of UTI is estimated to increase to 0.05 per person-year [8]. In men and women over 85 years old, the incidence of UTI increases substantially. A small cohort study in this age group found the incidence of UTI in women to be 0.13 per person-year and 0.08 per person-year in men [9].

*E. coli* is the most common organism responsible for causing UTIs in both community and healthcare settings, followed by other Enterobacteriaceae, such as *Proteus mirabilis, Klebsiella* spp.*,* and *Providencia *spp. Gram-positive organisms, such as methicillin-resistant *Staphylococcus aureus *and* Enterococcus*, are less common but frequently seen in healthcare settings and adults with chronic indwelling catheters [10,11]. Due to localized urinary symptoms and a characteristic clinical history, the clinical presentation of UTIs in older persons is more complicated than in young people.

Empirical antibiotics are prescribed for suspected UTIs in primary and secondary care settings, and more than 50% of these are unnecessary among elderly patients [12]. To reduce the use of antibiotics among the elderly, clinical and laboratory diagnosis of UTIs and sensitivity patterns are required. The incidence of UTIs in older persons is predicted to increase as our population ages, so diagnostic, treatment, and preventative methods must be improved to enhance their health.

This study aimed to assess the pattern of antibiotic sensitivity testing (AST) among bacteria that cause UTIs in elderly patients attending a tertiary care hospital based on urine culture samples. The study was conducted to compare the bacterial etiology and AST pattern of the bacteria causing UTI in elderly patients admitted in a tertiary care hospital, the intensive-care unit (ICU), the inpatient department (IPD), and the outpatient department (OPD). The association of the presence of comorbidities and outcomes associated with UTI were also analyzed in the study population.

## Materials and methods

Setting and data collection

This was a retrospective cross-sectional record-based study conducted in the Department of Microbiology of a tertiary care hospital in Bhubaneswar from January 2022 to December 2022. Permission was obtained from the appropriate authority to collect data. Data from the study period were collected and reviewed from electronic medical records (EMRs) and the Laboratory Information System. The study analyzed 4,030 urine samples that had been processed for urine culture. The study did not involve conducting new laboratory testing but relied entirely on previously documented results.

Sample collection and processing

As a routine, the samples collected from patients follow the said pattern; the OPD patients were advised to collect early morning clean catch midstream urine samples in a sterile wide-mouth container (per standard operating procedures) and send them to the microbiology laboratory. For IPD, noncatheterized patients were advised to collect the samples before initiating antimicrobial therapy or administering antibiotics. Urine samples were collected from catheter pipes for ICU and catheterized patients. After collection, samples were processed within one to two hours.

Processing of samples in the laboratory

Samples were processed in the microbiology laboratory following standard protocols. Urine samples were inoculated on cysteine lactose electrolyte-deficient medium and incubated aerobically for 24 hours or overnight at 37 °C. The isolated pathogens were identified and sensitivity-tested using an automated system (Vitek-2, bioMérieux, Marcy-l'Étoile, France). The different Vitek-2 antibiotic panel cards used were P628 for Gram-positive cocci, N235 for Gram-negative enterobacteria, N406 for Gram-negative nonfermenters, and YS08 for yeast for antifungal susceptibility testing. The AST results were interpreted according to the Clinical and Laboratory Standards Institute 2021.

Sample size and sampling technique

During the study period, 7,165 urine samples were received and processed for culture sensitivity, representing all study groups. Of these, 4,030 samples were from people over the 60-year age group, forming the final sample size of 4,030. The study used a convenience sampling technique.

Data analysis and statistical analysis

All data, demographic (age, gender, and place of patient in hospital), co-morbidities (diabetes mellitus, hypertension, HTN, and chronic kidney disease, CKD), and laboratory parameter (albumin) outcome of the patient (discharge, death or leave against medical advice), were collected from the EMRs and hospital database software and compiled using a Microsoft Excel spreadsheet (Microsoft Corporation, Redmond, WA). The data were entered into a Microsoft Excel spreadsheet, analyzed using Epi Info statistical software, version 7.2.3.1 (Centers for Disease Control and Prevention, Atlanta, GA), and presented as mean and standard deviation, frequencies, and percentages. Analysis of variance, chi-square test, and Fisher's exact test were used as tests of association, with a p value of <0.05 taken as statistically significant.

Ethical considerations

The study was approved by the Institutional Ethics Committee (KIIT/KIMS/IEC/1323/2023), dated 08.05.2023 before it was conducted.

## Results

A total of 4,030 urine samples were processed for urine culture during the study period, of which the number from each were ICU 1,600 (39.70%), OPD 1,175 (29.16%), and IPD 1,255 (31.14%). The maximum number (53.55%) of the elderly patients suffering from UTI belonged to 60-69 years: 797 from ICU, 673 from OPD, and 688 from IPD. The mean age of the study population suffering from clinical UTI was 69.74 ± 7.52 years, ranging from 60 to 97 years. The mean age of those admitted to the ICU with UTI was 70.56 ± 7.99, IPD patients was 68.97 ± 7.17, and OPD patients was 69.74 ± 7.52. The mean age of the study population admitted to the ICU was higher than those admitted to IPD and reporting to OPD; this difference was also found to be highly statistically significant (p < 0.0001). The total number of male patients was 2,559 (63.50%) and female patients was 1,471 (36.50%) (Table 1).

**Table 1:** Demographic characteristics of patients with UTI (n = 4,030) ^*^Applying ANOVA (Bartlett's test for inequality of population variances = 25.40; df = 2) ^**^Applying chi-square test; df = 2 ICU: intensive care unit, IPD: inpatient department, OPD: outpatient department; UTI: urinary tract infection; ANOVA: analysis of variance; df: degrees of freedom

Demographic variables	ICU (n = 1,600)	OPD (n = 1,175)	IPD (n = 1,255)	Total (n = 4,030)	p value
Mean age	70.56 ± 7.99	68.97 ± 7.17	69.41 ± 7.09	69.74 ± 7.52	<0.0001^*^
Age group (in years)					
60-69	797 (49.81%)	673 (57.28%)	688 (54.82%)	2,158	0.0003^**^
70-79	568 (35.50%)	393 (33.45%)	452 (36.02%)	1,413	0.3716^**^
≥80	235 (14.69%)	109 (9.28%)	115 (9.16%)	459	<0.0001^**^
Gender					
Male	1,029 (64.31%)	789 (62.87%)	741 (63.06%)	2,559	0.6805^**^
Female	571 (35.69%)	466 (37.13%)	434 (36.94%)	1,471	

About 81.61% (3,289 of 4,030) had type 2 diabetes mellitus, 78.91% (3,180 of 4,030) had HTN, and 42.21% (1,701 of 4,030) had CKD. The patients suffering from UTI and comorbidities were higher in ICU patients than in IPD and OPD patients. The association of comorbidities with UTI and admission to ICUs also showed a statistically significant association among hypertensive (p = 0.0269) and CKD patients (p = 0.0058). Around 33.39% (1,346 of 4,030) reported albumin in urine: 46.21% (622 of 1,346) from ICU, 23.48% (316 of 1,346) from OPD, and 30.31% (408 of 1,346) from IPD. Around 41.89% (1,688 of 4,030) reported a positive growth in culture: 33.94% (543 of those 1,600 in ICU) from ICU, 52.43% (616 of those 1,175 in OPD) from OPD, and 42.15% (529 of 1,255 of those from IPD) from IPD. Of the total patients, 69.13% (2,786 of 4,030 were discharged, 30.05% were cured, and 0.65% left against medical advice. The total number of deaths reported was seven (0.17%) (Table 2)*.*

**Table 2:** Medical characteristics of patients with UTI (n = 4,030) ^*^Applying chi-square test; df = 2 ^**^A total of 432 (21.28%) had insignificant and contaminants bacterial growth ^***^Applying Fisher’s exact test ICU: intensive care unit; IPD: inpatient department; OPD: outpatient department; type 2 DM: type 2 diabetes mellitus; LAMA: left against medical advice

Variables	ICU (n = 1,600)	OPD (n = 1,175)	IPD (n = 1,255)	Total (n = 4,030)	p value
Medical comorbidities					
Type 2 DM	1,301 (81.31%)	948 (80.68%)	1,040 (82.87%)	3,289	0.3517^*^
Hypertension	1,285 (80.31%)	896 (76.26%)	999 (79.60%)	3,180	0.0269^*^
Chronic kidney disease	717 (44.81%)	455 (38.72%)	529 (42.15%)	1,701	0.0058^*^
Lab parameters					
Albumin					
Absent	978 (61.13%)	859 (73.11%)	847 (67.49%)	2,684	<0.0001^*^
1+	472 (29.50%)	230 (19.57%)	312 (24.86%)	1,014	
2+	95 (5.94%)	59 (5.02%)	69 (5.50%)	223	
3+	54 (3.38%)	24 (2.04%)	26 (2.07%)	104	
4+	1 (0.06%)	3 (0.26%)	1 (0.08%)	5	
Bacterial culture^**^					
Growth positive	543 (33.94%)	616 (52.43%)	529 (42.15%)	1,688	<0.0001^*^
No growth	969 (60.56%)	377 (32.09%)	564 (44.94%)	1,910	
Outcome					
Discharged	1,578 (98.63)	8 (0.68%)	1,200 (95.62%)	2,786	<0.0001^*^
Cured	2 (0.13%)	1,167 (99.32%)	42 (3.35%)	1,211	<0.0001^*^
LAMA	13 (0.81%)	0 (0.00%)	13 (1.04%)	26	0.0035^*^
Death	7 (0.44%)	0 (0.00%)	0 (0.00%)	7	0.0049^***^

Total growth positive (1,688) comprised 1,560 bacterial isolates and 128 fungal isolates (*Candida* spp.). Of the total bacterial isolates (n = 1,560), *E. coli* was isolated in 39.67% (619/1,560), *Enterococcus faecalis* in 8.39% (131/1560), *Klebsiella* spp. in 20.70% (323/1,560), *S*. *aureus* in 5.64% (88/1,560), and *Pseudomonas aeruginosa* 3.78% (59/1,560) (Table 3).

**Table 3:** Cultural positivity with various organisms isolated (n = 1,560) OPD: outpatient department; IPD: inpatient department; ICU: intensive care unit

Organism	n = 1,560 (%)	ICU (n = 449) (%)	OPD (n = 646) (%)	IPD (n = 465) (%)
E. coli	619 (39.67%)	143 (9.16%)	295 (18.91%)	181 (11.60%)
K. pneumonia	323 (20.70%)	100 (6.41%)	107 (6.85%)	116 (7.43%)
Pseudomonas aeruginosa	59 (3.78%)	19 (1.21%)	23 (1.47%)	17 (1.08%)
E. faecalis	131 (8.39%)	41 (2.62%)	42 (2.69%)	48 (3.07%)
S. aureus	88 (5.64%)	14 (0.89%)	40 (2.56%)	34 (2.17%)
Other Enterobacterales (*Enterobacter* spp., *Citrobacter* spp., *Klebsiella* spp., *Serratia* spp., *Proteus*, *Providencia*, and *Morganella* spp.)	93 (5.96%)	32 (2.05%)	43 (2.75%)	18 (1.15%)
Others	247 (15.83%)	100 (6.41%)	96 (6.15%)	51 (3.26%)

The highest percentage, 47.65% (295/619), of* E. coli* was isolated from OPD patients. Other bacteria, such as *Enterobacter *spp.*, Serratia *spp.*, Citrobacter *spp.*, *and the *Proteae tribe, *were isolated in 5.96% (93/1,560) of cases.

**Figure 1:**
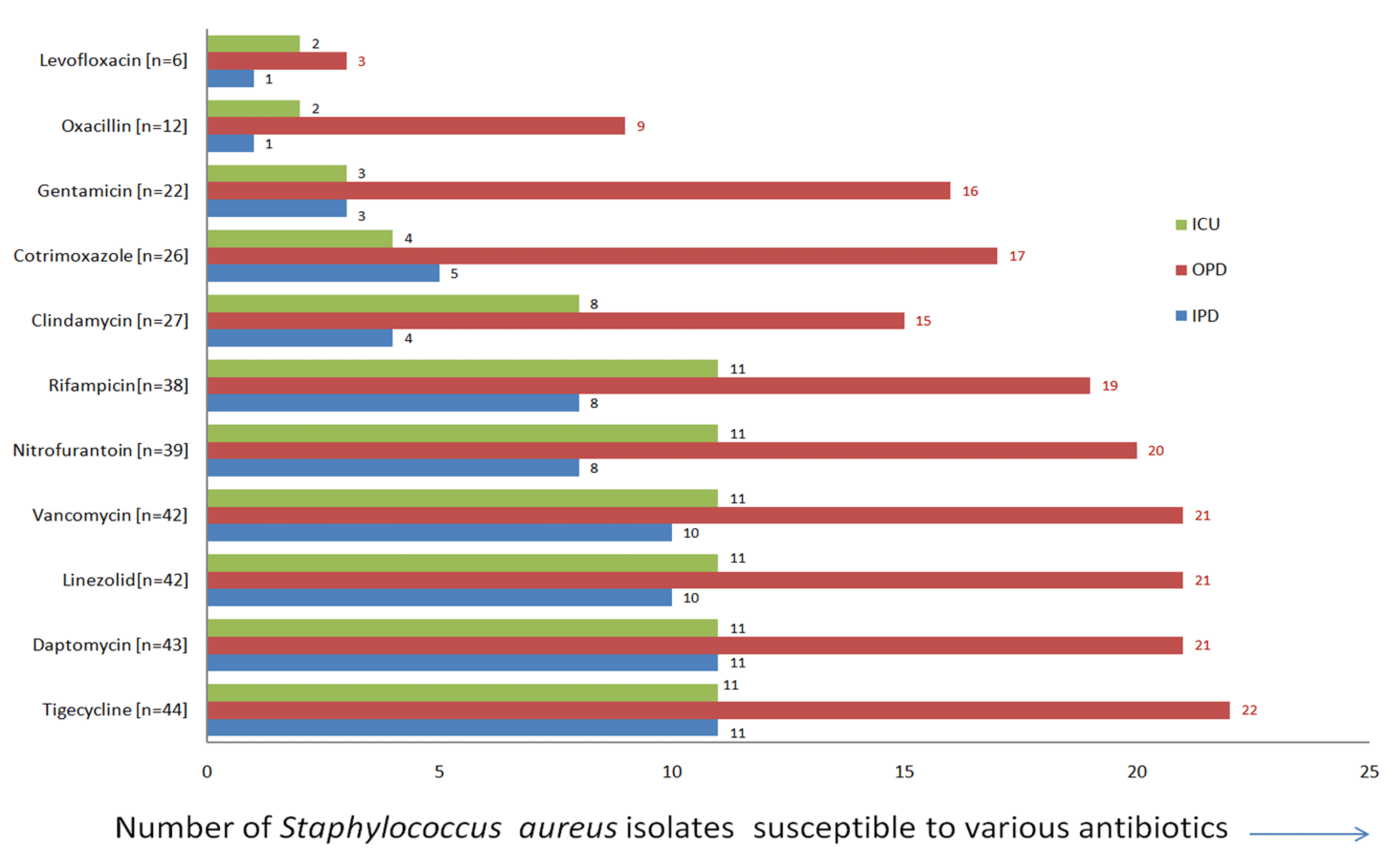
Antibiotic sensitivity (in numbers) graph of S. aureus (n = 88) OPD: outpatient department; IPD: inpatient department; ICU: intensive care unit

*S. aureus* accounted for a total of 5.64% (88 of 1,560) of bacterial isolates. Those isolates, isolated from OPD patients, were sensitive to most antibiotics: tigecycline (22/44), daptomycin (21/43), linezolid (21/42), vancomycin (21/42), nitrofurantoin (20/39), rifampicin (19/38), clindamycin (15/27), cotrimoxazole (17/26), and gentamicin (16/22). *S. aureus* isolated from ICU and IPD patients was least sensitive to antibiotics, as depicted in Figure 1.

**Figure 2:**
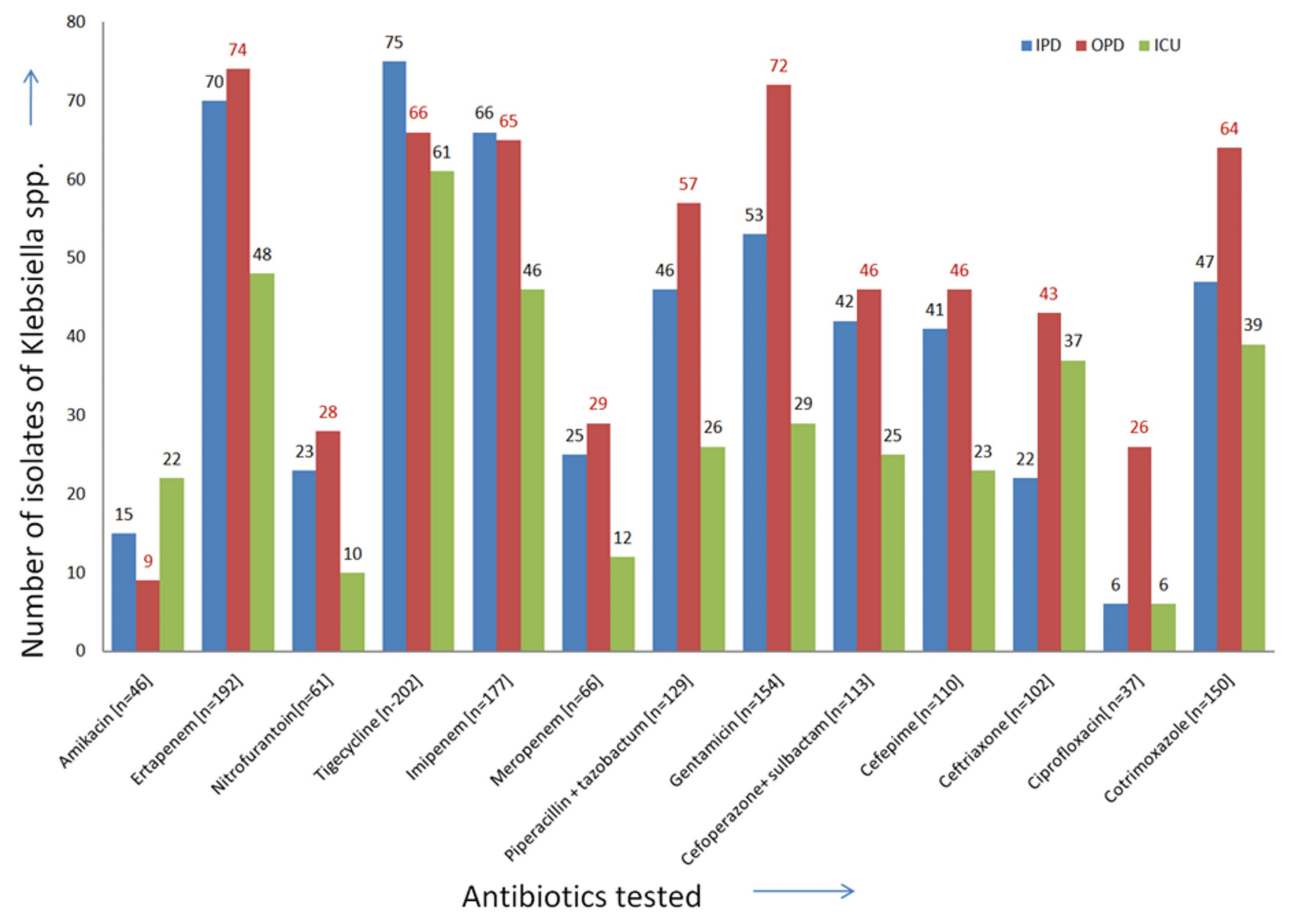
Antibiotic sensitivity (in numbers) graph of Klebsiella spp. (n = 326) ICU: intensive care unit; IPD: inpatient department; OPD: outpatient department

*Klebsiella* spp*. *accounted for 20.70% (323/1,560) of the bacterial isolates. It showed a similar pattern of sensitivity to antibiotics in both OPD and IPD patients and was sensitive mainly to ertapenem, tigecycline, and gentamicin (Figure 2).

**Figure 3:**
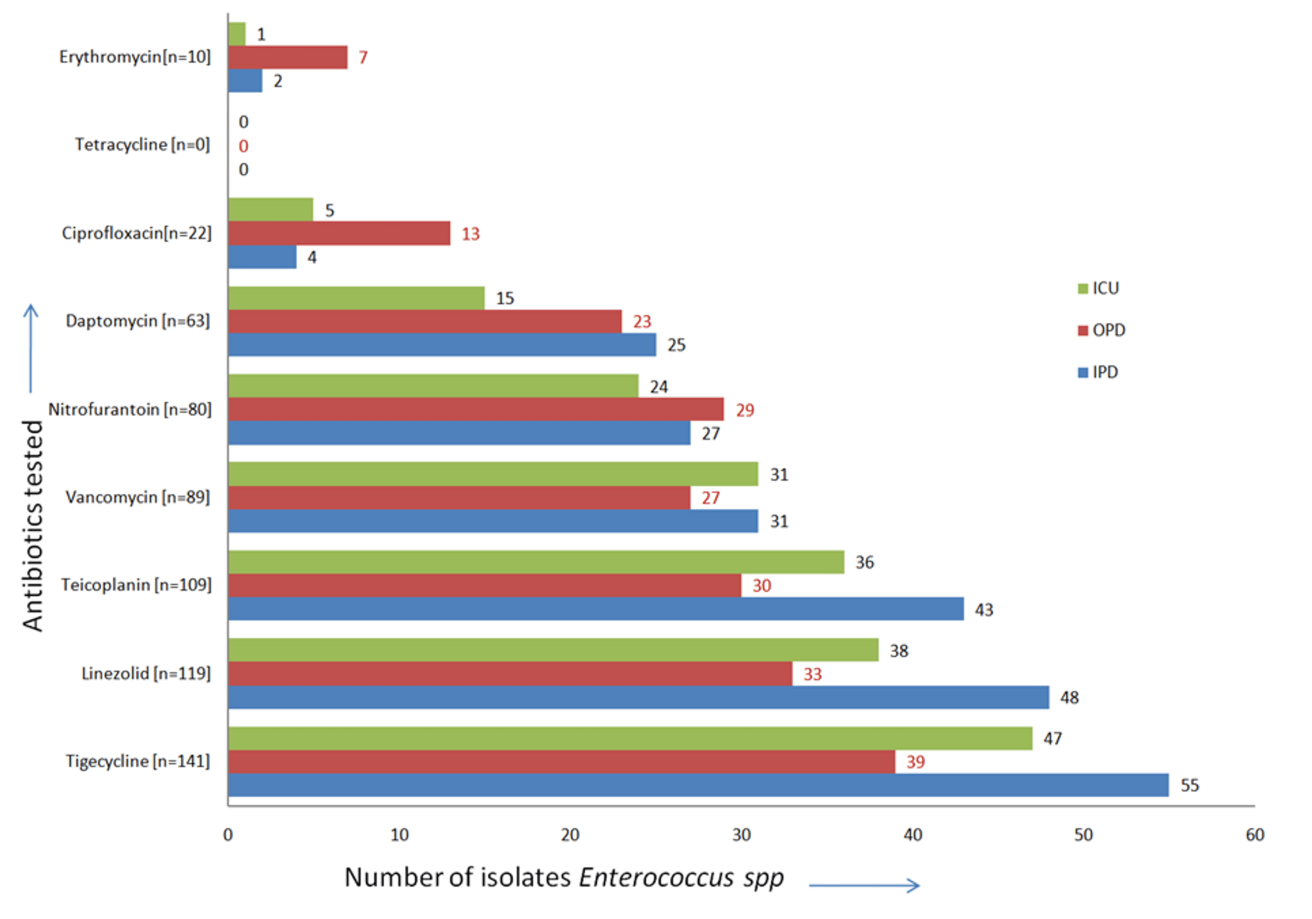
Antibiotic sensitivity (in numbers) graph of E. faecalis (n = 160) OPD: outpatient unit; IPD: inpatient department; ICU: intensive care unit

*E. faecalis* showed a similar pattern of sensitivity to all the antibiotics. It was mainly sensitive to tigecycline, linezolid, teicoplanin, and vancomycin isolated from the study population (Figure 3).

**Figure 4:**
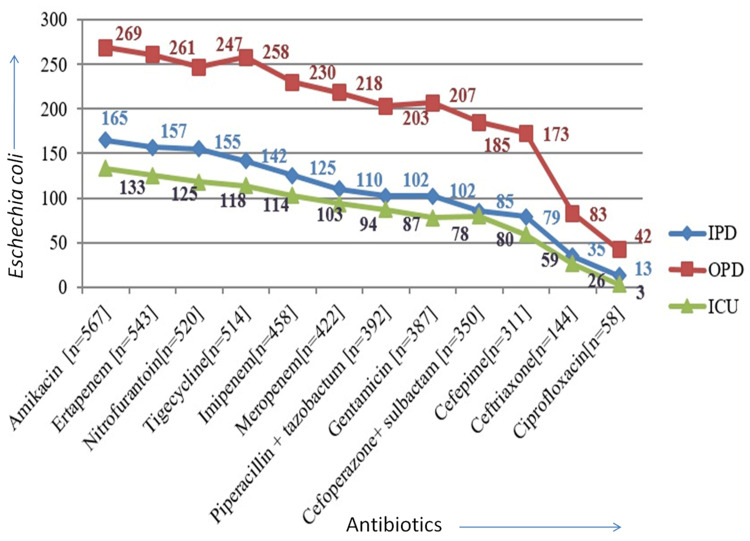
Antibiotic sensitivity (in numbers) graph of E. coli (n = 619) OPD: outpatient department; IPD: inpatient department; ICU: intensive care unit

*E. coli* isolated from OPD patients showed the highest sensitivity to most antibiotics compared to IPD and ICU patients. Amikacin was the highest sensitivity in* E. coli*, followed by ertapenem, nitrofurantoin, tigecycline, meropenem, piperacillin, tazobactam, gentamicin, cefoperazone, sulbactam, cefepime, ceftriaxone, and ciprofloxacin (Figure 4).

## Discussion

This study compares the bacterial etiology and AST of elderly patients admitted with UTI, with a distribution in three different settings: the ICU, IPD, and OPD of a tertiary care hospital in Eastern India. It also throws light on the association of comorbidities and the outcomes of patients with UTI.

The prevalence of UTI was higher (53.54%) in the 60-69-year-old population in the current study. Other studies have also shown that the incidence of UTI rises in patients over the age of 65 in comparison with patients of middle age [13,14]. Another study by Yoshikawa et al. showed that UTI was an important cause of morbidity and sepsis in elderly patients, having a spectrum varying from benign cystitis to potentially life-threatening pyelonephritis [15]. Another study by Curns et al. showed that around 16% of hospital admissions in patients over the age of 65 were due to UTIs [16]. A similar finding is also recorded in a study by Ezz et al. [17]. In contrast to our findings, studies by Patel and Pattani have mentioned that the prevalence rate of UTI was higher in the age group of 70-79 years (60.4%) [18].

The mean age of the study population suffering from clinical UTI was 69.74 ± 7.52 years, similar to a study by Swamy et al., in which the mean age was 69.4 ± 7.37 years [19]. Our study showed a higher prevalence of UTI in male patients (63.49%) than in female patients (36.51%), which differs from the findings of other studies, which revealed that the frequency of UTI was greater in female patients than male patients [20,21].

Similar to the results of the other two research studies, the authors have stated findings similar to the current study, wherein the presence of comorbidities with UTIs was higher among those in ICUs [22,23]. In the current study, diabetes and HTN were the most common morbidities among all three groups: ICU, OPD, and IPD. In this study, of all UTI cases, 82% of cases have a history of diabetes mellitus, while studies conducted by Ezz et al. and Parveen et al. revealed that 64%-70% had diabetes mellitus [17,24]. The mortality rate in our study was lower (0.17%), and the discharge rate was close (69%) to that of a study by Kakde et al. in which the mortality rate was higher (29.16%), and the discharge rate was 71% [25].

*E. coli* and *Klebsiella pneumoniae* are the most common Gram-negative pathogens causing UTI in our study, which was like the findings of Alpay et al. [26]. *E. coli* isolates were susceptible to carbapenems (88%), as in a study by Kakde et al. [25].

The percentage of bacteria isolated from ICU, IPD, and OPD patients varied. The highest isolation percentage was from OPD patients (47.66%), followed by 29.24% from IPD and 23.10% from ICU. Similar variations in the isolation of *E. coli* among IPD and OPD patients were observed in another study [27]. In our study, *K. pneumoniae* isolates were mostly resistant to amikacin (86%), which contradicted the findings by Kakde et al. [25].

*S. aureus* isolated from OPD patients was more sensitive to antibiotics than IPD and ICU patients in the elderly population. IPD and ICU patients showed similar patterns of AST to *S. aureus*. It was most sensitive to tigecycline, daptomycin, linezolid, vancomycin, and nitrofurantoin. It was moderately sensitive to rifampicin, clindamycin, cotrimoxazole, and gentamicin. The organism was resistant to levofloxacin. Similar findings were observed by Fontana et al. [28].

*Enterococcus *spp. was the most common Gram-positive pathogen in our study, like the study by Parveen et al. [24]. *Enterococcus* spp. causes UTI mostly in children <10 years old and adults older than 60, mainly due to more frequent anatomical defects in the urinary tract and long-term catheterization [17,21].* Enterococci* are characterized by intrinsic resistance to some beta-lactam antibiotics (cephalosporins, meropenem), which is related to their structure's lack of appropriate penicillin-binding proteins (PBP). Furthermore, *Enterococcus *spp. may overproduce modified PBP5 proteins having low affinity for beta-lactams and conferring resistance to ampicillin and amoxicillin, as suggested by Fontana et al. [28].

In the current study, *E. faecalis* was mainly sensitive to tigecycline, linezolid, teicoplanin, and vancomycin, which were isolated from the study population. It was also sensitive to erythromycin, ciprofloxacin, and daptomycin isolated from OPD patients. Similar findings were also observed in article; *E. faecalis* showed a high rate of resistance to gentamicin and streptomycin high level, while it was susceptible to ampicillin (96.7%), ampicillin/sulbactam (99.4%), imipenem (98.3%), linezolid (99.4%), nitrofurantoin (99.6%), teicoplanin (98.5%), tigecycline (98.9%), and vancomycin (98.2%). Similar findings were found in the article [29].

Limitations

There are certain limitations in the current study. First, as the study is a retrospective data analysis, it is plausible that the clinical data and outcomes we omitted were influenced by confounding variables. Second, as this is a single-center study, its findings could not be applied to different contexts. Furthermore, a considerable portion of patients with incomplete medical data were eliminated, which could potentially reduce the significance of our results. Finally, due to a lack of information in the medical records, we were unable to gather data on adverse events linked to antibiotics or the doctors' adherence to recommendations.

## Conclusions

UTIs and ASB are highly prevalent in older adults. Overuse of antibiotics for ASB remains a significant problem, coupled with the challenge faced by clinicians in distinguishing symptomatic UTIs from ASB. High rates of antibiotic resistance and a wide range of bacterial pathogens limit therapeutic options. Also, individual strategies and comorbidities should be considered in the treatment of geriatric patients. As drug resistance among bacterial pathogens changes over time and place, regular surveillance and monitoring are essential to provide physicians with updated information on the most compelling empirical treatment of UTIs among elderly patients. Empirical antibiotic choice in treating UTIs should be based on the knowledge of the local prevalence of causative microorganisms and their antibiogram rather than on universal guidelines.
